# The interplay of personality traits, anxiety, and depression in Chinese college students: a network analysis

**DOI:** 10.3389/fpubh.2023.1204285

**Published:** 2023-08-03

**Authors:** Tianqi Yang, Zhihua Guo, Xia Zhu, Xufeng Liu, Yaning Guo

**Affiliations:** ^1^Section of Basic Psychology, Department of Military Medical Psychology, Air Force Medical University, Xi'an, Shaanxi, China; ^2^Section of Military Psychology, Department of Military Medical Psychology, Air Force Medical University, Xi'an, Shaanxi, China

**Keywords:** personality, anxiety, depression, college students, network analysis

## Abstract

**Background:**

Anxiety and depression are among the greatest contributors to the global burden of diseases. The close associations of personality traits with anxiety and depression have been widely described. However, the common practice of sum scores in previous studies limits the understanding of the fine-grained connections between different personality traits and anxiety and depression symptoms and cannot explore and compare the risk or protective effects of personality traits on anxiety and depression symptoms.

**Objective:**

We aimed to determine the fine-grained connections between different personality traits and anxiety and depression symptoms and identify the detrimental or protective effects of different personality traits on anxiety and depression symptoms.

**Methods:**

A total of 536 college students from China were recruited online, and the average age was 19.98 ± 1.11. The Chinese version of the Ten-Item Personality Inventory, Generalized Anxiety Disorder-7, and Patient Health Questionnaire-9 was used to investigate the personality traits and symptoms of anxiety and depression of participants after they understood the purpose and filling method of the survey and signed the informed consent. The demographic characteristics were summarized, and the scale scores were calculated. The network model of personality traits and symptoms of anxiety and depression was constructed, and bridge expected influence (BEI) was measured to evaluate the effect of personality traits on anxiety and depression. The edge accuracy and BEI stability were estimated, and the BEI difference and the edge weight difference were tested.

**Results:**

In the network, 29 edges (indicating partial correlations between variables) bridged the personality community and the anxiety and depression community, among which the strongest correlations were extraversion-fatigue, agreeableness-suicidal ideation, conscientiousness-uncontrollable worry, neuroticism-excessive worry, neuroticism-irritability, and openness-feelings of worthlessness. Neuroticism had the highest positive BEI value (0.32), agreeableness had the highest negative BEI value (−0.27), and the BEI values of neuroticism and agreeableness were significantly different from those of most other nodes (*p* < 0.05).

**Conclusion:**

There are intricate correlations between personality traits and the symptoms of anxiety and depression in college students. Neuroticism was identified as the most crucial risk trait for depression and anxiety symptoms, while agreeableness was the most central protective trait.

## 1. Introduction

Anxiety and depression are among the greatest contributors to the global burden of diseases (1), with detrimental effects on mental and physical health, such as an increased risk of suicidal thoughts and suicide attempts (2, 3), insomnia (4), daily maladaptive health behaviors (5), Parkinson’s disease, and cardiovascular disease (6, 7). In particular, early adulthood — college undergraduate students are usually in this stage — is a critical period, with heightened susceptibility to anxiety and depressive disorders, with more than 20% of young adults meeting the criteria for anxiety disorders and the prevalence of depression reaching 25% among undergraduate students (8, 9). Recent studies showed that the prevalence of anxiety and depression among Chinese college students was also high (10–13), for example, at 22.7 and 46.8%, respectively, according to a study (13). In addition to common consequences, college students who have developed anxiety or depression may have difficulties in academic functioning and suffer from low quality of life, heightening the necessity for research into this issue in China.

Given that anxiety and depression are prevalent and often accompanied by a decline in quality of life, unpleasant symptoms, and impaired social relationships, it is important to identify the pathogenesis of anxiety and depression. Personality traits have been identified as risk/protective factors for anxiety and depression (9, 14–18). The Big Five (i.e., five facets of personality traits including neuroticism, extraversion, agreeableness, conscientiousness, and openness) is one of the most relevant frameworks when examining the personality trait related to anxiety and depression (17, 19, 20). As the Big Five represents personality traits that usually precede the symptoms of anxiety and depression, they are considered risk/protective factors for anxiety and depression (20, 21). A cross-sectional study on Taiwan college students found that neuroticism was significantly positively associated with anxiety and depression scores while agreeableness was significantly inversely associated with anxiety and depression scores (21). Another study also revealed that agreeableness was inversely associated with depression while neuroticism was significantly associated with depression scores (17). Studies have reported that high levels of conscientiousness and extraversion were protective against the deleterious effects of high levels of neuroticism on depressive mood (22). Consistently, a systematic review showed that neuroticism was considered a risk factor while extraversion and conscientiousness were protective factors for affective disorders such as depression and anxiety (20).

As described above, the relationships between the Big Five personality traits and anxiety and depression have been extensively investigated. Many researchers have used total scores to measure anxiety and depression when investigating the relationships between the Big Five and anxiety and depression, assuming that anxiety or depression is a holistic psychological construct. For example, a previous study used the standardized Depression Anxiety Stress Scale-21 (DASS-21) to assess anxiety and depression and five subscales of the Big Five Inventory (BFI) to assess the Big Five, then examined the associations between the DASS-21 total score and the Big Five (21). According to the cumulative risk hypothesis, the overall risk of a negative outcome, such as depression and anxiety, is magnified by interaction with distinct personality traits (23). For instance, openness can moderate the influence of extraversion, and these two personality traits work together to reduce the risk of anxiety (24). In addition to the fact that personality involves the interaction of different traits, depression and anxiety are subsumptions containing different interacting symptoms (25). However, considering that anxiety and depressive disorders consist of distinct symptoms, the common practice of sum scores, according to previous studies (26, 27), obscures the relative importance of different symptoms and limits the understanding of the fine-grained connections between different personality traits and anxiety and depression symptoms. Imagine two individuals have the same sum score, they would commonly be considered to have the same degree of depression; however, one may have a high score on anhedonia and a low score on fatigue while the other may demonstrate the reverse pattern; their depression actually differs according to the relative importance of anhedonia (core symptom) and fatigue (28). Hence, analysis at a symptom level may provide a way forward, which is essential to understanding psychopathological pathways and effective intervention targets that could not be discovered by relying solely on total scores (27, 29, 30). To the best of our knowledge, there is a paucity of data on symptom-level links between personality traits and symptoms of anxiety and depression, which hampers the identification of more efficacious targets to intervene. This knowledge gap motivated the present study.

One approach to achieve the study objectives is network analysis, which is a data-driven method used to reveal the connections among individual variables, regardless of whether these variables are symptoms (31–35). In the network theory, psychopathological constructs are represented and visualized as networks emerging from interactions between distinct variables, which indicates that the variables (symptoms or otherwise) and their active interactions lead to the development and maintenance of constructs (32, 33, 36). The network commonly consists of nodes, which represent variables, and edges, representing correlations between variables (37). Network analysis can yield important findings. It can be used to examine the fine-grained relationships between individual variables (38–40), shedding light on the important psychopathological pathways between constructs via edge weights. The approach also identifies bridge nodes, which connect to nodes of another community composed of a theory-based group of variables and are key regarding the impacts of one community on another; the identified bridge variables can be considered promising and effective targets for prevention, intervention, and treatment (40–43).

Network analysis has been used to study the relationships between personality traits and psychopathological constructs. For example, a study examined the network structure of substance use disorder, the Big Five personality traits, impulsivity, and psychopathological constructs, including depression and anxiety; however, the presence of anxiety or depression was the sole node in its community, which did not reveal the symptom-level relationships between personality traits and depression and anxiety (44). In another previous study, the network structure of schizotypal personality, autistic traits, obsessive–compulsive traits, depression, and anxiety was investigated; however, this analysis also regarded depression or anxiety as merely one node in the network rather than as a symptom-level construct (45). To the best of our knowledge, there is a lack of network analysis studies investigating the symptom-level relationships of the Big Five personality traits with symptoms of anxiety and depression.

To fill this knowledge gap, we constructed a network consisting of the Big Five personality traits, anxiety symptoms, and depression symptoms. We aimed to elucidate the important pathways linking personality traits with anxiety and depression and to identify important bridge nodes that maximally link nodes among different communities. Based on the findings, we attempted to provide theoretical insights into the specific pathways between distinct personality traits and individual anxiety or depression symptoms and provide implications for prevention and intervention in light of the risk and protective roles that personality traits play.

## 2. Materials and methods

### 2.1. Participants

We designed the online survey powered by www.wjx.cn, and the systematically trained investigators sent the quick response code of the survey to the WeChat group of students from three colleges in Xi’an and Shanghai, China. We explained the purpose and the filling method of the survey and obtained the informed consent of the participants. From April to May 2022, 536 students completed the survey. The Chinese version of the Ten-Item Personality Inventory (TIPI-C), Generalized Anxiety Disorder-7 (GAD-7), and Patient Health Questionnaire-9 (PHQ-9) was used to investigate the personality traits and symptoms of anxiety and depression of college students. Questionnaires that were not fully answered were excluded. A total of 507 (94.59%) questionnaires were valid. This study strictly followed the tenets of the Declaration of Helsinki and was approved by the Ethics Committee of the Xijing Hospital of the Air Force Medical University (KY20224106-1).

### 2.2. Measures

#### 2.2.1. Tipi-c

The TIPI-C is the Chinese version of a scale developed by Li (46) based on the TIPI (47) to assess personality traits, and the reliability and validity of the scale meet the demand of psychometrics for measuring the personality traits of Chinese people (46). It is a short scale with 10 items and five factors, namely, neuroticism, conscientiousness, agreeableness, openness, and extraversion. The scale uses a 7-point Likert scale, ranging from one (completely disagree) to seven (completely agree).

#### 2.2.2. Gad-7

The GAD-7 was compiled by Spitzer et al. (48), as a self-screening tool to assess anxiety symptoms. The Chinese version of GAD-7 used for this study was revised by He et al. (49), and it has good reliability and validity in the Chinese population (49). It contains seven items scored on a 4-point Likert scale, from zero (not at all) to three (almost every day). The GAD-7 total score ranges from zero to 21, and a higher score represents severer anxiety symptoms. The GAD-7 had high reliability in this study (Cronbach’s α coefficient = 0.90).

#### 2.2.3. PHQ-9

The PHQ-9 was developed by Kroenke et al. (50) to measure the severity of depression in the past 2 weeks. The Chinese version of PHQ-9 (51) was used in this study, and it has been proven to have good reliability and validity among Chinese adolescents (52). The scale contains nine items scored on a 4-point Likert scale from zero (not at all) to three (almost every day). The total score of the PHQ-9 ranges from zero to 27, and the higher the score the severer the depression symptoms. Cronbach’s α coefficient of the PHQ-9 in this study was 0.88.

### 2.3. Statistical analysis

SPSS 22.0 was used to summarize the demographic characteristics of participants and calculate the scale scores. R 4.1.1 software was used to construct the network model, measure bridge centrality, and test the robustness of the network.

#### 2.3.1. Network model construction

The R package qgraph (53) was used for network model construction. In the network, nodes represented dimensions of the TIPI-C and items of the GAD-7 and PHQ-9. The term “community” in network analysis is used to indicate a theory-based group of nodes that correspond to a psychological structure or psychiatric disorder (40). The nodes in this study were divided into two communities, namely, the personality community and the anxiety and depression community. Edge in the network represented a partial correlation of two nodes after statistical control for the interference of other nodes (54). The least absolute shrinkage and selection operator (LASSO) regularization (55) and extended Bayesian information criterion (EBIC) (56) were used in combination to obtain a comprehensible network by setting trivial edges to a weight of zero (37). We set the EBIC hyperparameter γ to 0.5 and used the Fruchterman-Reingold (57) algorithm to lay out the network.

#### 2.3.2. Bridge centrality measurement

The R package network tools were used in the bridge centrality measurement (40). The bridge expected influence (BEI) was used in this study given its suitability for networks with positive and negative edges (58). The BEI of a node is defined as the sum of the edge weights between the node and nodes from another community; the higher the BEI value of a node the stronger the influence of the node on the other community (58).

#### 2.3.3. Network robustness test

The R package bootnet was used to test the robustness of the network (37). The non-parametric bootstrapping method (1,000 bootstrapped samples) was used to estimate the 95% confidence intervals of edge weights to test the edge accuracy. Case-dropping bootstrapping (1,000 bootstrapped samples) was used to test the BEI stability, and the correlation stability (CS) coefficient was calculated to quantify the stability. Ideal stability is indicated by a CS coefficient higher than 0.5 (37). The bootstrapping method (1,000 bootstrapped samples) was used to test the BEI difference of nodes and the edge weight difference of node pairs in the network (*α* = 0.05).

## 3. Results

### 3.1. Demographic characteristics and descriptive statistics

Demographic characteristics of the college students are shown in Table 1. The means, standard deviations, and BEI values of nodes in the network are shown in Table 2.

**Table 1:** Demographic characteristics of the college students (*N* = 507).

**Variable**	Mean (SD), range, and constituent ratio
*Age*	19.98 (1.11), 16–24
*Sex*	
Male	170, 33.53%
Female	337, 66.47%
*Only child*	161, 31.76%
*Developmental environment*	
Rural	176, 34.71%
Urban	331, 65.29%
*Single parent*	59, 11.64%

**Table 2:** The means, standard deviations, and BEI values of nodes in the personality trait–anxiety and depression network.

Nodes	Abbreviation	M	SD	BEI
*Personality traits*				
Extraversion	EXT	4.26	1.19	−0.12
Agreeableness	AGR	4.90	1.10	−0.27
Conscientiousness	CON	4.33	1.12	−0.14
Neuroticism	NEU	3.63	1.14	0.32
Openness	OPE	4.43	1.10	−0.08
*Anxiety symptoms*				
Feeling nervous, anxious, or on edge	Nervousness or anxiety	0.74	0.78	0.00
Not being able to stop or control worrying	Uncontrollable worry	0.69	0.84	−0.07
Worrying too much about different things	Excessive worry	0.74	0.82	0.14
Trouble relaxing	Trouble relaxing	0.60	0.79	−0.01
Being so restless that it is hard to sit still	Restlessness	0.53	0.76	−0.05
Becoming easily annoyed or irritable	Irritability	0.72	0.84	0.11
Feeling afraid as if something awful might happen	Fear that something might happen	0.58	0.80	−0.05
*Depression symptoms*				
Little interest or pleasure in doing things	Anhedonia	0.95	0.81	−0.07
Feeling down, depressed, or hopeless	Depressed or sad mood	0.87	0.74	0.00
Trouble falling/staying asleep or sleeping too much	Sleep difficulties	0.90	0.90	0.02
Feeling tired or having little energy	Fatigue	0.89	0.80	−0.05
Poor appetite or overeating	Appetite changes	0.77	0.85	0.00
Feeling bad about yourself—or that you are a failure or have let yourself or your family down	Feelings of worthlessness	0.76	0.78	−0.09
Trouble concentrating on things, such as reading the newspaper or watching television	Concentration difficulties	0.67	0.84	−0.05
Moving or speaking so slowly that other people could have noticed, or the opposite— moving around a lot more than usual, being fidgety or restless	Psychomotor agitation/retardation	0.59	0.79	−0.03
Thoughts that you would be better off dead or hurting yourself in some way	Suicidal ideation	0.48	0.80	−0.09

### 3.2. The personality trait–anxiety and depression network structure

**Figure 1:**
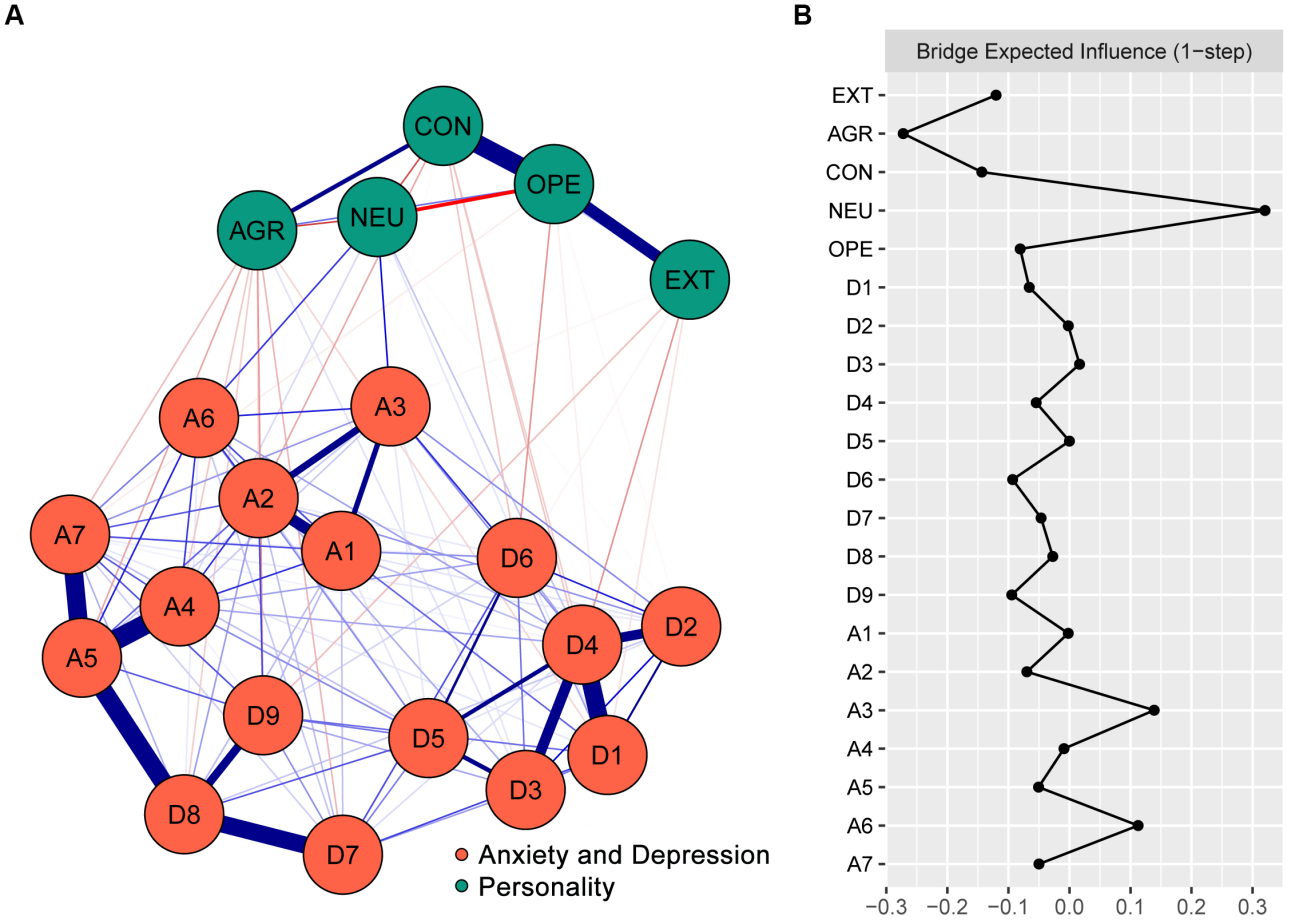
The structure of the personality trait–anxiety and depression network among college students and the BEI values of nodes. **(A)** The structure of the personality trait–anxiety and depression network. Blue edges represent positive partial correlations, and red edges represent negative partial correlations. The wider the edge, the stronger the partial correlation. **(B)** The BEI values of nodes in the network (raw scores). EXT, Extraversion; AGR, Agreeableness; CON, Conscientiousness; NEU, Neuroticism; and OPE, Openness. A1, nervousness or anxiety; A2, uncontrollable worry; A3, excessive worry; A4, trouble relaxing; A5, restlessness; A6, irritability; A7, fear that something might happen; D1, anhedonia; D2, depressed or sad mood; D3, sleep difficulties; D4, fatigue; D5, appetite changes; D6, feelings of worthlessness; D7, concentration difficulties; D8, psychomotor agitation/retardation; D9, suicidal ideation.

Figure 1A displays the structure of the personality trait–anxiety and depression network. The network contained 125 non-zero edges (edge weights ranged from-0.15 to 0.23), among which 29 edges bridged the personality community and the anxiety and depression community (23.20%). Of these cross-community edges, EXT was negatively correlated with A7 “fear that something might happen,” D1 “anhedonia,” D4 “fatigue,” D7 “concentration difficulties,” and D9 “suicidal ideation”; the strongest correlation was with D4 “fatigue” (edge weight = −0.06). AGR was positively correlated with D3 “sleep difficulties” (edge weight = 0.02) and negatively correlated with eight nodes of the anxiety and depression community, among which the strongest correlation was with D9 “suicidal ideation” (edge weight = −0.06). CON was negatively correlated with A1 “nervousness or anxiety,” A2 “uncontrollable worry,” D1 “anhedonia,” D4 “fatigue,” and D6 “feelings of worthlessness”; the strongest correlation with CON was A2 “uncontrollable worry” (edge weight = −0.05). NEU was positively correlated with six nodes of the anxiety and depression community, among which the strongest correlations were with A3 “excessive worry” (edge weight = 0.14) and A6 “irritability” (edge weight = 0.11). OPE was negatively correlated with A7 “fear that something might happen,” D1 “anhedonia,” D2 “depressed or sad mood,” and D6 “feelings of worthlessness”; the strongest correlation was with D6 “feelings of worthlessness” (edge weight = −0.06). The correlation matrix of the network is displayed in Supplementary Table S1 of the Supplementary Material.

As shown in Supplementary Figure S1 of the Supplementary Material, the relatively narrow 95% CIs of edge weights indicated that these edge weight estimations were accurate. The results of difference tests on edge weights are shown in Supplementary Figure S2 of the Supplementary Material.

### 3.3. Bei values of nodes in the network

Figure 1B shows the BEI values of the nodes in the personality trait–anxiety and depression network. In the personality community, NEU had the highest positive BEI value (0.32), and AGR had the highest negative BEI value (−0.27). In the anxiety and depression community, A3 “excessive worry” had the highest BEI value (0.14).

As shown in Supplementary Figure S3 in the Supplementary Material, the BEI values of NEU, AGR, and A3 “excessive worry” were significantly different from the BEI values of most other nodes (*p* < 0.05). Supplementary Figure S4 in the Supplementary Material displays the results of the BEI stability test. The CS coefficient of BEI in the network was 0.67, suggesting ideal stability.

## 4. Discussion

There are two prevailing views on the relationship between mental disorders and symptoms. The perspective of classification diagnosis considers symptoms to reflect mental disorders, as in the DSM-5 (32). The dimension diagnosis perspective considers mental disorders as a compound of different symptom dimensions (59). However, both of these perspectives overlook the interactions between symptoms, a fundamental phenomenon in mental disorders (60). According to the network theory, mental disorders are a dynamic system composed of interacting symptoms (61); in the network structure, the edges that bridge communities reveal the psychopathological interactions among different psychological structures and mental disorders (62). As the nodes in the network belong to two different communities measuring personality traits and symptoms of anxiety and depression respectively, the nodes within a community have high consistency; the connections within a community are closer than those across the community. However, the understanding of crucial cross-community edges is based on the network theory of the relationship between mental disorders and symptoms, which can enhance our knowledge of the fine-grained relationships between personality traits and anxiety and depression symptoms. In view of this, we discussed the strongest cross-community edges in the network.

Among symptoms correlated with extraversion, “fatigue” had the strongest negative edge weight. Fatigue is a general feeling of weariness and lack of energy and motivation (63). Extroversion is often accompanied by low allostatic load and great aerobic capacity (64), and high extraversion is correlated with better health outcomes and well-being, such as lower frailty, fewer affective symptoms, and fewer sleeping problems (65–68); thus, individuals high in extraversion are prone to experience low levels of fatigue. Indeed, the advantages of extraversion may explain the positive effects of cognitive behavioral therapy in chronic fatigue syndrome, alleviating daily fatigue and pain, both mentally and physically (68). “Suicidal ideation” was negatively correlated with agreeableness. As one of the most empirically tested theories, the interpersonal-psychological theory of suicide provides a theoretical framework that explains this relationship. The theory assumes that there are three factors underpinning suicidal thoughts and behaviors, namely, perceived burdensomeness, thwarted belongingness, and acquired capability for suicide (the degree to which one is able to enact suicide attempts) (69). Perceived burdensomeness is an individual’s belief that they are a burden to their family, friends, or society, and thwarted belongingness is an unmet need for social connection. Suicidal ideation increases when perceived burdensomeness and thwarted belongingness cooccur (70); however, this cooccurrence is less likely in individuals with high agreeableness, who tend to be good-natured, modest, cooperative, and emotionally satisfied by social interaction (71). Conscientiousness was negatively correlated with “uncontrollable worry” in college students. Conscientiousness is characterized by individual differences in the propensity for self-discipline, orderliness, and reliability in the pursuit of work completion (72). According to Gao et al. (73), self-control is a key component in the structure of conscientiousness, and individuals with high conscientiousness are adept at controlling unnecessary worries. Neuroticism was positively correlated with “irritability” and “excessive worry.” Neuroticism is defined as emotional negativity and instability; people high in neuroticism are irrational perfectionists and tend to catastrophize difficulties (74); they are more prone to irritability when encountering setbacks (75). A correlation between neuroticism and worry was previously reported (76), and the neural basis of excessive worry helps explain how neuroticism contributes to psychopathological vulnerability (77). Openness was negatively correlated with “feelings of worthlessness.” From a cognitive perspective, individuals who tend to seek, comprehend, and utilize complex patterns of information in the world might be more adaptable and therefore less susceptible to depression (78). However, the results of multiple meta-analyzes have revealed no direct association between openness and depression (79, 80). The contradictory results may stem from complicated associations between openness and specific symptoms of depression; the non-correlation between openness and depression on an overall level covered up the internal fine-grained relationships.

BEI reflects the role of a given variable in maintaining the interaction between different psychological structures or mental disorders (81); variables with high BEI values can be regarded as bridges and serve as potential targets to reduce or enhance this interactive influence (58, 81). In the present study, BEI value helped to identify the detrimental or protective effects of different personality traits on anxiety and depression symptoms. Neuroticism was identified as the most crucial risk trait for depression and anxiety symptoms, while agreeableness was the most central protective trait according to their highest positive and negative BEI values, respectively. Neuroticism reflects emotional instability and is a powerful predictor of negative emotional experiences, including depression and anxiety (82–84). A previous study suggested that neuroticism and depression share a common genetic basis (85). A plausible neural mechanism underlying the vulnerability of individuals high in neuroticism to depression is sensitivity to stress-related reductions in response of the ventral striatum to reward (86). Individual differences in neuroticism and trait anxiety were predicted by volume variation in the left amygdala (18). The negative correlations between agreeableness and anxiety and between agreeableness and depression have been confirmed by many studies (87, 88). Individuals high in agreeableness are prone to positive emotions, and agreeableness has been found to be a protective factor against anxiety induced by the COVID-19 pandemic (89). From a network perspective, compared with other intervening personality traits, reducing neuroticism and enhancing agreeableness have more advantages in reducing anxiety and depression. In addition, digital applications have been proven to be an effective method for personality intervention (90).

There are many studies on the relationship between the different Big Five personality traits and anxiety and depression (22, 91–95). However, these studies treated anxiety or depression as a whole and overlooked the interconnections of different symptoms. In addition, due to the limitations of statistical methods, the risk or protective effects of different personality traits on anxiety and depression cannot be compared. Based on network analysis, this study provides us with a clear understanding of the risk or protective effects of each personality trait on different anxiety and depression symptoms and helps quantitatively compare the overall risk or protective effects of different personality traits on anxiety and depression, thus providing a reference for potential intervention targets.

Several limitations should be noted when interpreting the findings of the current study. First, a cross-sectional design was used; thus, the directionality and causality of the relationship between personality traits and symptoms of anxiety and depression could not be determined (96). Second, the study used a convenience sample of Chinese college students, potentially limiting external validity and generalization to the broader population. Third, TIPI-C is a brief scale, and the facets of the personality traits TIPI-C covered may not be as sufficient as other Big Five personality scales. Last, the selection of psychological intervention targets was based on the network analysis theory; relatively weak effects of the relationships between personality traits and symptoms of anxiety and depression may indicate limited practical application of the results, and further intervention research is needed to verify whether interventions targeting the identified bridge variables will be effective.

In summary, the present study represents the first utilization of network analysis to elucidate the relationship between personality traits and anxiety and depression in college students. The intuitive network models help to develop a comprehensive understanding of the fine-grained correlations of personality traits with anxiety and depression. BEI values facilitated the identification of the key variables bridging personality traits with anxiety and depression and highlighted potential targets for psychological intervention.

## Data availability statement

The raw data supporting the conclusions of this article will be made available by the authors, without undue reservation.

## Ethics statement

The studies involving human participants were reviewed and approved by The Ethics Committee of the Xijing Hospital of the Air Force Medical University (KY20224106-1). Written informed consent to participate in this study was provided by the participants or their legal guardian/next of kin.

## Author contributions

YG, XL, and XZ: concept and design and critical revision of the manuscript. TY and ZG: acquisition of data and drafting of the manuscript. ZG and TY: analysis and interpretation of the data. All authors contributed to the article and approved the submitted version.

## Funding

This study was funded by the Air Force Medical University (KJZFJ2020-1, 2021JQ-335).

## Conflict of interest

The authors declare that the research was conducted in the absence of any commercial or financial relationships that could be construed as a potential conflict of interest.

## Publisher’s note

All claims expressed in this article are solely those of the authors and do not necessarily represent those of their affiliated organizations, or those of the publisher, the editors and the reviewers. Any product that may be evaluated in this article, or claim that may be made by its manufacturer, is not guaranteed or endorsed by the publisher.

## Abbreviations

TIPI-C: Chinese version of the Ten-Item Personality Inventory
GAD-7: Generalized Anxiety Disorder-7
PHQ-9: Patient Health Questionnaire-9
LASSO: least absolute shrinkage and selection operator
EBIC: extended Bayesian information criterion
BEI: bridge expected influence
CS coefficient: correlation stability coefficient
DSM: Diagnostic and Statistical Manual of Mental Disorders
