# Supplementary material for: The interplay of personality traits, anxiety, and depression in Chinese college students: a network analysis
Source: Front Public Health. 2023 Aug 3;11:1204285. doi: 10.3389/fpubh.2023.1204285 (PMC10434527; doi:10.3389/fpubh.2023.1204285)
Supplement: Supplementary file 1 [file Table_1.DOCX]

Table S1 The [Correlation](javascript:;) [Matrix](javascript:;) of the Personality-Anxiety and Depression Network in Chinese College Students

|  | EXT | AGR | CON | NEU | OPE | D1 | D2 | D3 | D4 | D5 | D6 | D7 | D8 | D9 | A1 | A2 | A3 | A4 | A5 | A6 | A7 |
| --- | --- | --- | --- | --- | --- | --- | --- | --- | --- | --- | --- | --- | --- | --- | --- | --- | --- | --- | --- | --- | --- |
| EXT |  | 0.0000 | 0.0311 | 0.0000 | 0.1933 | -0.0165 | 0.0000 | 0.0000 | -0.0596 | 0.0000 | 0.0000 | -0.0050 | 0.0000 | -0.0357 | 0.0000 | 0.0000 | 0.0000 | 0.0000 | 0.0000 | 0.0000 | -0.0033 |
| AGR | 0.0000 |  | 0.1539 | -0.1094 | 0.0821 | -0.0226 | 0.0000 | 0.0166 | 0.0000 | 0.0000 | 0.0000 | -0.0414 | -0.0273 | -0.0588 | 0.0000 | -0.0211 | 0.0000 | -0.0302 | -0.0505 | 0.0000 | -0.0369 |
| CON | 0.0311 | 0.1539 |  | -0.0987 | 0.2272 | -0.0322 | 0.0000 | 0.0000 | -0.0274 | 0.0000 | -0.0334 | 0.0000 | 0.0000 | 0.0000 | -0.0019 | -0.0486 | 0.0000 | 0.0000 | 0.0000 | 0.0000 | 0.0000 |
| NEU | 0.0000 | -0.1094 | -0.0987 |  | -0.1512 | 0.0141 | 0.0010 | 0.0000 | 0.0326 | 0.0000 | 0.0000 | 0.0000 | 0.0000 | 0.0000 | 0.0000 | 0.0000 | 0.1388 | 0.0213 | 0.0000 | 0.1125 | 0.0000 |
| OPE | 0.1933 | 0.0821 | 0.2272 | -0.1512 |  | -0.0086 | -0.0028 | 0.0000 | 0.0000 | 0.0000 | -0.0597 | 0.0000 | 0.0000 | 0.0000 | 0.0000 | 0.0000 | 0.0000 | 0.0000 | 0.0000 | 0.0000 | -0.0096 |
| D1 | -0.0165 | -0.0226 | -0.0322 | 0.0141 | -0.0086 |  | 0.1426 | 0.0738 | 0.2239 | 0.0000 | 0.0497 | 0.1080 | 0.0000 | 0.0830 | 0.0870 | 0.0000 | 0.0000 | 0.0000 | 0.0000 | 0.0007 | 0.0139 |
| D2 | 0.0000 | 0.0000 | 0.0000 | 0.0010 | -0.0028 | 0.1426 |  | 0.1391 | 0.1815 | 0.0162 | 0.1257 | 0.0000 | 0.0325 | 0.0000 | 0.0161 | 0.0602 | 0.0658 | 0.0000 | 0.0000 | 0.0000 | 0.0124 |
| D3 | 0.0000 | 0.0166 | 0.0000 | 0.0000 | 0.0000 | 0.0738 | 0.1391 |  | 0.1862 | 0.1547 | 0.0810 | 0.0571 | 0.0000 | 0.0508 | 0.0000 | 0.0000 | 0.0141 | 0.0666 | 0.0000 | 0.0461 | 0.0355 |
| D4 | -0.0596 | 0.0000 | -0.0274 | 0.0326 | 0.0000 | 0.2239 | 0.1815 | 0.1862 |  | 0.1528 | 0.0477 | 0.0196 | 0.0000 | 0.0000 | 0.0390 | 0.0000 | 0.0623 | 0.0511 | 0.0000 | 0.0526 | 0.0066 |
| D5 | 0.0000 | 0.0000 | 0.0000 | 0.0000 | 0.0000 | 0.0000 | 0.0162 | 0.1547 | 0.1528 |  | 0.1449 | 0.0712 | 0.0980 | 0.0773 | 0.0147 | 0.0248 | 0.0140 | 0.0542 | 0.0000 | 0.0644 | 0.0000 |
| D6 | 0.0000 | 0.0000 | -0.0334 | 0.0000 | -0.0597 | 0.0497 | 0.1257 | 0.0810 | 0.0477 | 0.1449 |  | 0.1008 | 0.0000 | 0.0241 | 0.0616 | 0.0126 | 0.1189 | 0.0538 | 0.0000 | 0.0000 | 0.0101 |
| D7 | -0.0050 | -0.0414 | 0.0000 | 0.0000 | 0.0000 | 0.1080 | 0.0000 | 0.0571 | 0.0196 | 0.0712 | 0.1008 |  | 0.2195 | 0.0626 | 0.0360 | 0.0375 | 0.0000 | 0.0000 | 0.0000 | 0.0316 | 0.0268 |
| D8 | 0.0000 | -0.0273 | 0.0000 | 0.0000 | 0.0000 | 0.0000 | 0.0325 | 0.0000 | 0.0000 | 0.0980 | 0.0000 | 0.2195 |  | 0.1734 | 0.0384 | 0.0556 | 0.0000 | 0.0000 | 0.2249 | 0.0120 | 0.0445 |
| D9 | -0.0357 | -0.0588 | 0.0000 | 0.0000 | 0.0000 | 0.0830 | 0.0000 | 0.0508 | 0.0000 | 0.0773 | 0.0241 | 0.0626 | 0.1734 |  | 0.0405 | 0.1049 | 0.0000 | 0.0067 | 0.0933 | 0.0000 | 0.0973 |
| A1 | 0.0000 | 0.0000 | -0.0019 | 0.0000 | 0.0000 | 0.0870 | 0.0161 | 0.0000 | 0.0390 | 0.0147 | 0.0616 | 0.0360 | 0.0384 | 0.0405 |  | 0.1899 | 0.1590 | 0.1173 | 0.0000 | 0.1040 | 0.1002 |
| A2 | 0.0000 | -0.0211 | -0.0486 | 0.0000 | 0.0000 | 0.0000 | 0.0602 | 0.0000 | 0.0000 | 0.0248 | 0.0126 | 0.0375 | 0.0556 | 0.1049 | 0.1899 |  | 0.1667 | 0.1165 | 0.0893 | 0.1126 | 0.0944 |
| A3 | 0.0000 | 0.0000 | 0.0000 | 0.1388 | 0.0000 | 0.0000 | 0.0658 | 0.0141 | 0.0623 | 0.0140 | 0.1189 | 0.0000 | 0.0000 | 0.0000 | 0.1590 | 0.1667 |  | 0.0289 | 0.0298 | 0.1253 | 0.0587 |
| A4 | 0.0000 | -0.0302 | 0.0000 | 0.0213 | 0.0000 | 0.0000 | 0.0000 | 0.0666 | 0.0511 | 0.0542 | 0.0538 | 0.0000 | 0.0000 | 0.0067 | 0.1173 | 0.1165 | 0.0289 |  | 0.2336 | 0.1133 | 0.0941 |
| A5 | 0.0000 | -0.0505 | 0.0000 | 0.0000 | 0.0000 | 0.0000 | 0.0000 | 0.0000 | 0.0000 | 0.0000 | 0.0000 | 0.0000 | 0.2249 | 0.0933 | 0.0000 | 0.0893 | 0.0298 | 0.2336 |  | 0.1244 | 0.2268 |
| A6 | 0.0000 | 0.0000 | 0.0000 | 0.1125 | 0.0000 | 0.0007 | 0.0000 | 0.0461 | 0.0526 | 0.0644 | 0.0000 | 0.0316 | 0.0120 | 0.0000 | 0.1040 | 0.1126 | 0.1253 | 0.1133 | 0.1244 |  | 0.0714 |
| A7 | -0.0033 | -0.0369 | 0.0000 | 0.0000 | -0.0096 | 0.0139 | 0.0124 | 0.0355 | 0.0066 | 0.0000 | 0.0101 | 0.0268 | 0.0445 | 0.0973 | 0.1002 | 0.0944 | 0.0587 | 0.0941 | 0.2268 | 0.0714 |  |

Note: EXT = Extraversion, AGR = Agreeableness, CON = Conscientiousness, NEU = Neuroticism, OPE = Openness, A1 = nervousness or anxiety; A2 = uncontrollable worry; A3 = excessive worry; A4 = trouble relaxing; A5 = restlessness; A6 = irritability; A7 = fear something might happen; D1 = anhedonia; D2 = depressed or sad mood; D3 = sleep difficulties; D4 = fatigue; D5 = appetite changes; D6 = feelings of worthlessness; D7 = concentration difficulties; D8 = psychomotor agitation/retardation; D9 = suicidal ideation.
