# Supplementary figures and images for: The interplay of personality traits, anxiety, and depression in Chinese college students: a network analysis
Source: Front Public Health. 2023 Aug 3;11:1204285. doi: 10.3389/fpubh.2023.1204285 (PMC10434527; doi:10.3389/fpubh.2023.1204285)

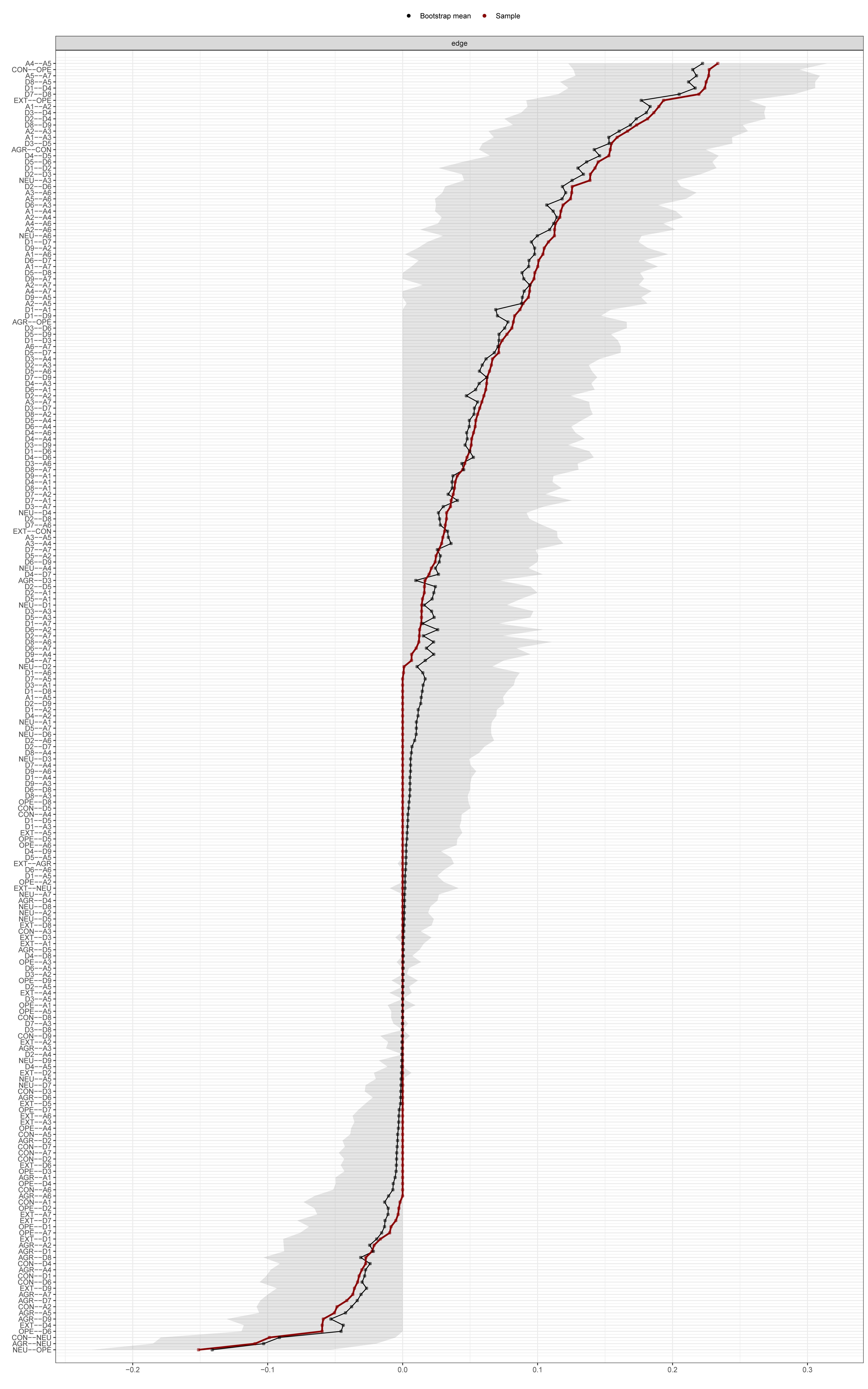

Supplement: Supplementary file 2 [file Image_1.TIF]

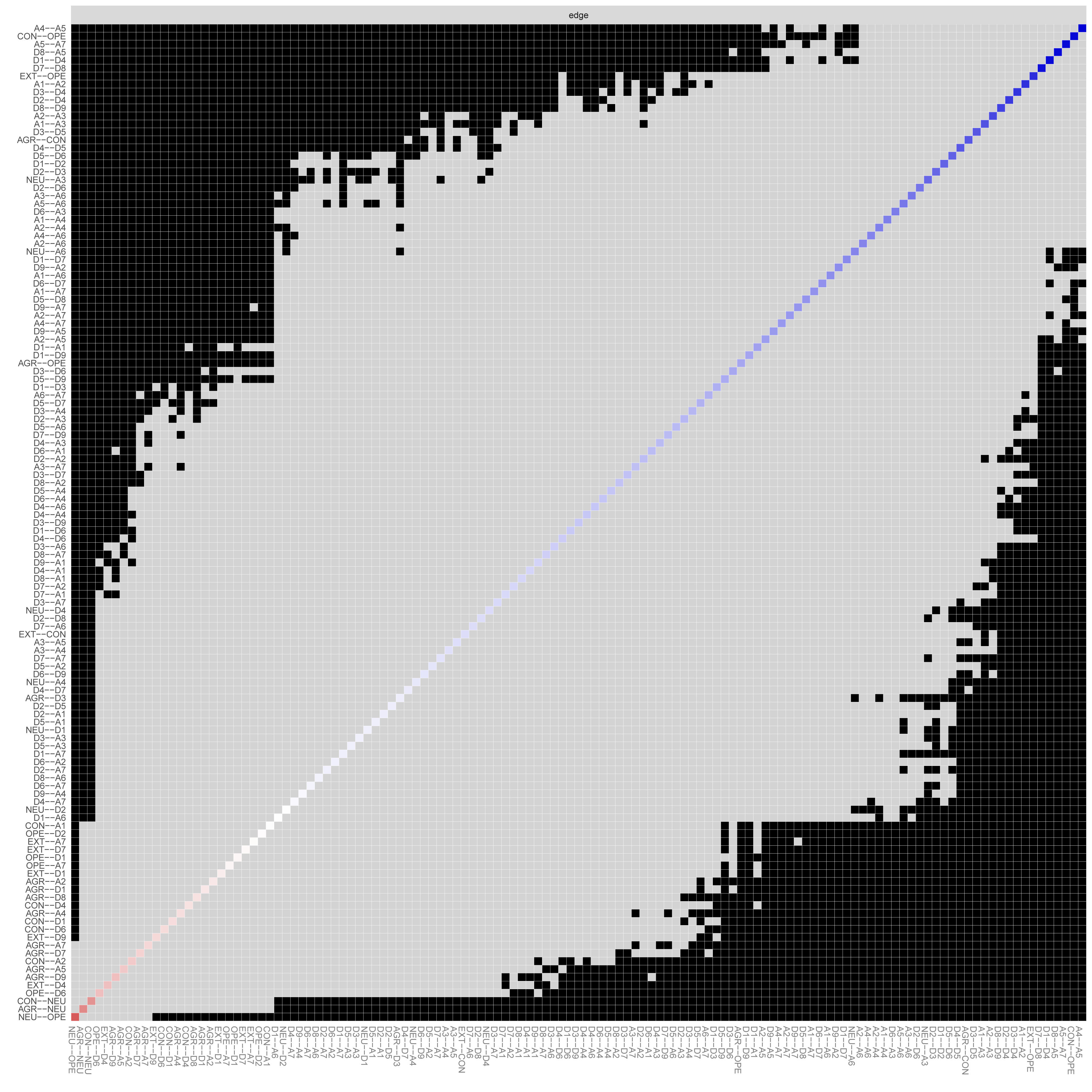

Supplement: Supplementary file 3 [file Image_2.TIF]

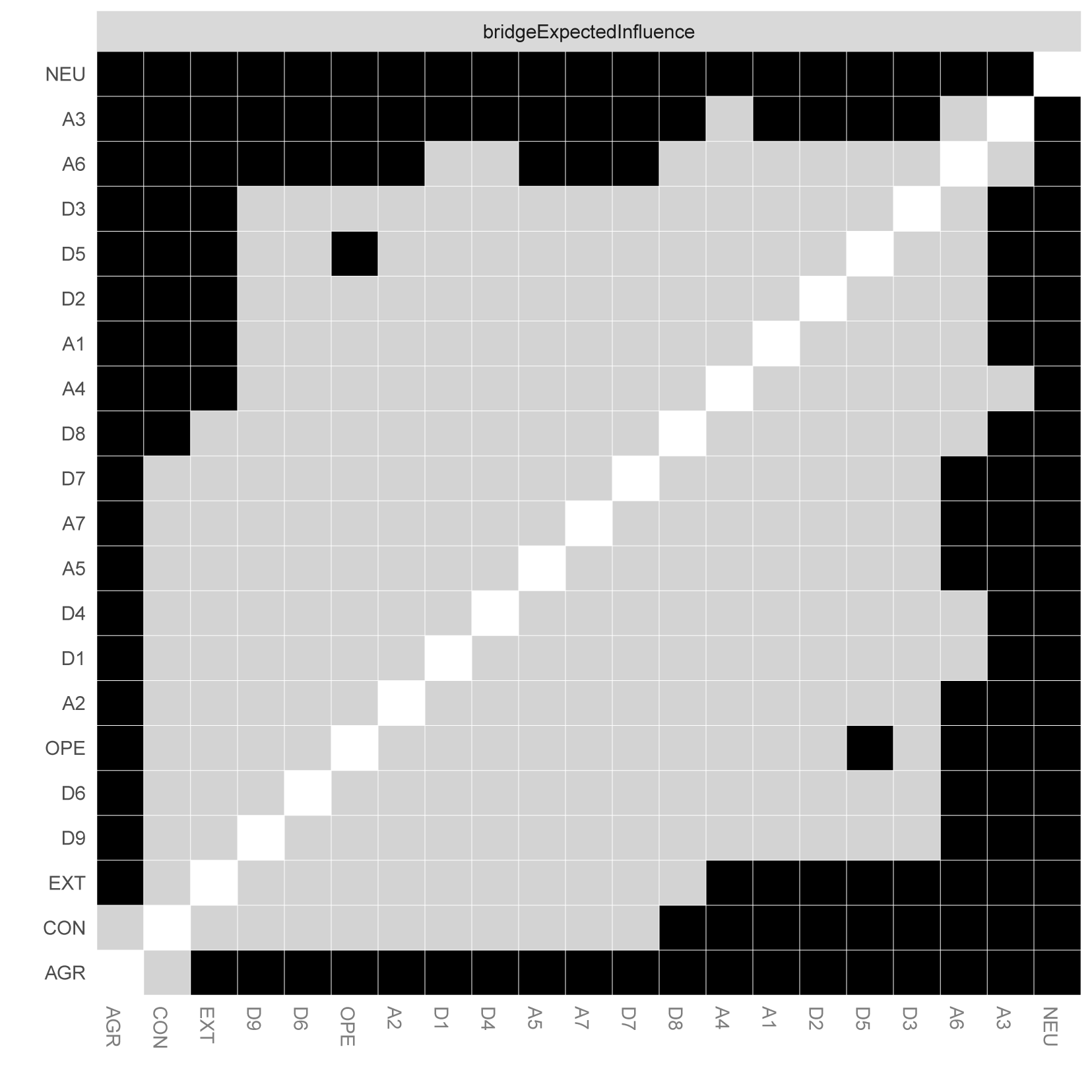

Supplement: Supplementary file 4 [file Image_3.TIF]

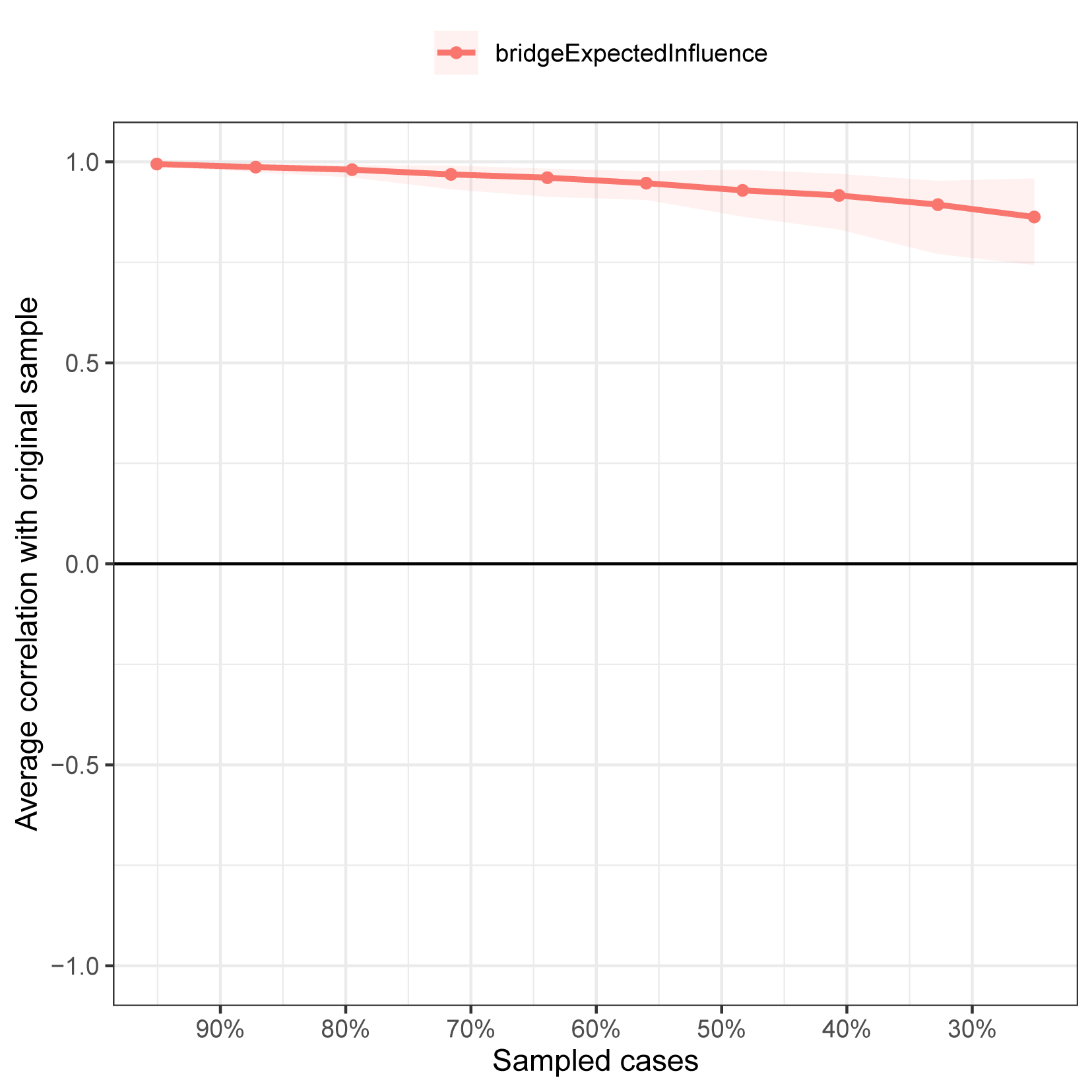

Supplement: Supplementary file 5 [file Image_4.TIF]
